# Intra-Articular Cytokine Levels in Adolescent Patients after Anterior Cruciate Ligament Tear

**DOI:** 10.1155/2018/4210593

**Published:** 2018-08-28

**Authors:** Marco Bigoni, Marco Turati, Giovanni Zatti, Marta Gandolla, Paola Sacerdote, Massimiliano Piatti, Alberto Castelnuovo, Luca Rigamonti, Daniele Munegato, Silvia Franchi, Nicola Portinaro, Alessandra Pedrocchi, Robert J. Omeljaniuk, Vittorio Locatelli, Antonio Torsello

**Affiliations:** ^1^Orthopedic Department, San Gerardo Hospital, Monza, Italy; ^2^School of Medicine and Surgery, University of Milano-Bicocca, Monza, Italy; ^3^Department of Paediatric Orthopaedic Surgery, Hospital Couple Enfant, Grenoble Alpes University, Grenoble, France; ^4^Department of Electronics, Information and Bioengineering, NearLab, Politecnico di Milano, Milan, Italy; ^5^Department of Pharmacological and Biomolecular Sciences, University of Milan, Milan, Italy; ^6^Department of Paediatric Orthopaedics and Neuro-Orthopaedics, Humanitas Research Hospital, University of Milan, Rozzano, Milan, Italy; ^7^Department of Biology, Lakehead University, Thunder Bay, ON, Canada P7B5E1

## Abstract

The treatment of anterior cruciate ligament (ACL) injuries in children and adolescents is challenging. Preclinical and clinical studies investigated ACL repairing techniques in skeletally immature subjects. However, intra-articular bioenvironment following ACL tear has not yet been defined in skeletally immature patients. The aim of this study was to measure cytokine concentrations in the synovial fluid in adolescent population. Synovial levels of IL-1*β*, IL-1ra, IL-6, IL-8, IL-10, and TNF-*α* were measured in 17 adolescent patients (15 boys) with ACL tears who underwent ACL reconstruction including acute (5), subacute (7), and chronic (5) phases. Femoral growth plates were classified as “open” in three patients, “closing” in eight, and “closed” in six. Eleven patients presented an ACL tear associated with a meniscal tear. The mean Tegner and Lysholm scores (mean ± SD) of all patients were 8 ± 1 and 50.76 ± 26, respectively. IL-8, TNF-*α*, and IL-1*β* levels were significantly greater in patients with “open” physes. IL-1ra and IL-1*β* levels were significantly higher in patients with ACL tear associated with a meniscal tear. Poor Lysholm scores were associated with elevated IL-6 and IL-10 levels. IL-10 levels positively correlated with IL-6 and IL-8 levels, whereas TNF-*α* concentration negatively correlated with IL-6 levels. Skeletally immature patients with meniscal tears and open growth plates have a characteristic cytokine profile with particularly elevated levels of proinflammatory cytokines including IL-8, TNF-*α*, and IL-1*β*. This picture suggests that the ACL tear could promote an intra-articular catabolic response in adolescent patients greater than that generally reported for adult subjects. The study lacks the comparison with synovial samples from healthy skeletally immature knees due to ethical reasons. Overall, these data contribute to a better knowledge of adolescent intra-articular bioenvironment following ACL injuries.

## 1. Introduction

The increased participation of children and adolescents (skeletally immature) in sports has increased the number of sport-related injuries [1]. To illustrate, a recent epidemiologic study of American high school athletes showed that the knee is the most common severely injured site (29%), with particular incidence of ligament tears (45.4%) [2]. An analysis of American insurance data revealed that 6.7% of all injuries are anterior cruciate ligament (ACL) tears in 5–18-year-old soccer players. Moreover, ACL injuries have significantly increased over the last years in the 11–18-year age range [3].

Ideal treatment of ACL tears in skeletally immature patients is still controversial. Surgical intervention risks could result in growth arrest and asymmetric epiphyseal growth; this possibility, consequently, may delay intervention until the patient nears skeletal maturity [4, 5]. Surgical reconstruction is generally recognized to have advantages over noninvasive treatment, but it has a very high failure rate. Noninvasive treatment of patients with open epiphyses is generally avoided since it could require the adolescent to completely stop playing sports. As well, unintended consequences of delayed surgical treatment of ACL deficiency include chondral degeneration, subsequent meniscal lesions, and early onset of osteoarthritis (OA) [6–8].

Although ACL reconstruction restores joint stability and reduces the risk of meniscal and chondral lesions, the effect of surgery on OA progression remains unclear. In a 12-year follow-up study, 103 female soccer players (average age 19 years) presented a high incidence of knee OA following ACL tear and reconstruction (51%) compared with the contralateral knee (8%) [9].

Such injuries stimulate large increases in synovial fluid catabolic species, which contribute to progressive cartilage destruction [10–12]. High levels of proinflammatory interleukin-8 (IL-8), interleukin-6 (IL-6), and tumor necrosis factor-*α* (TNF-*α*) and low levels of protective interleukin-10 (IL-10) and interleukin-1 receptor antagonist (IL-1ra) were described in synovial fluid of adults with acute or chronic ACL tears, supporting the important role of these species in posttraumatic OA [13–16]. By contrast, the synovial environment following an ACL tear in skeletally immature patients remains largely undescribed.

The healing potential of the anterior cruciate ligament is among the most prominent and controversial topics in orthopaedic research. The feasibility of maintaining the native ACL tissue compared to the success of the available arthroscopic reconstruction techniques is the object of an interesting debate [17–19]. A confounding factor is that the pediatric population has different musculoskeletal characteristics compared to adults [20–22]. In particular, experimental models have shown a higher ACL functional healing in skeletally immature experimental animals with a more productive response to ACL transaction [19, 23]. Skeletally immature patients are characterized by a high biological ACL cellular activity [24], but the intra-articular inflammatory environment after an ACL tear has not yet been defined in this population.

The aim of this study was to assess the concentration profiles of relevant knee synovial cytokines (IL-1*β*, IL-1ra, IL-6, IL-8, IL-10, and TNF-*α*) in an injured adolescent population relative to time from trauma, growth plate maturity, and presence or absence of meniscal tear.

## 2. Methods

### 2.1. Subjects

**Figure 1:**
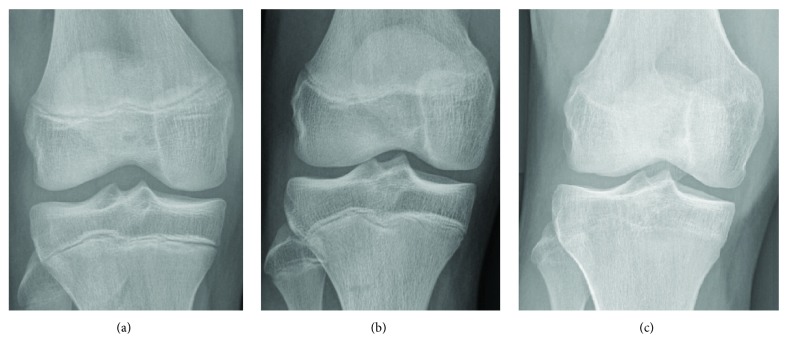
Growth plates classification: (a) open physis, (b) closing physis, and (c) closed physis.

Seventeen patients (15 males and 2 females; aged 15.76 ± 1.52 years) participated in this retrospective study performed between 2005 and 2011. Inclusion criteria were (i) age less than eighteen years, (ii) a Tanner scale ranging from 3 to 4, and (iii) attestation of ACL tear by a senior orthopaedic surgeon based on physical examination, radiographs, MRI of the knee, and arthroscopic examination. Exclusion criteria were (i) previous history of knee infection, injury, or surgery; (ii) inflammatory or arthritic diseases; (iii) systemic inflammation at the time of the collection of the sample; (iv) posterior cruciate lesion; (v) physical rehabilitation programs involving the injured knee; and (vi) previous intra-articular injection of any drugs. Growth plate maturity was assessed on anterior-posterior knee radiographs. Femoral growth plates were classified into three types including (i) open, (ii) closing, and (iii) closed (Figure 1) [25]. A physis was defined closing when areas of open and closed physes were both observed in the radiographs [26].

During arthroscopic surgery, the same senior orthopaedic surgeon assessed chondral and meniscal lesions. Chondral status was evaluated using the Outerbridge scoring system: (0) normal cartilage, (I) cartilage softening, (II) cartilage fibrillation or fissuring involving <1.25 cm area, (III) fissures involving >1.25 cm area, and (IV) subchondral bone exposed [27].

All patients were clinically evaluated using the Lysholm score and the Tegner activity scale at the time of the arthrocentesis [28]. The Lysholm score ranges from 0 to 100 points and is a scale which defines to what extent knee pain has effects in the ability to manage everyday life. A score higher than 95 is considered to be excellent, 84 to 94 is good, 65 to 83 is fair, and less than 65 is poor [29]. The Tegner activity scale evaluates patients' activity levels before the knee injury with a numerical scale ranging from an activity level of 0 (sick leave due to knee problems) to 10 (competitive sports on a very high level).

Protocols were approved by the local Human Research Ethical Committee and conformed to the principles outlined in the WMA Declaration of Helsinki. Both parents of each patient signed a written informed consent.

### 2.2. Samples

Arthrocentesis of the knee was aseptically performed without lavage at the time of the first evaluation or at the beginning of the arthroscopic surgery. Due to ethical reasons, we were not authorised by the Ethical Committee to obtain synovial fluid from the contralateral uninjured knee. After centrifugation at 3000*g*, synovial fluid was collected in tubes containing EDTA and stored at −80°C until assayed [15, 30]. Samples were analyzed for interleukin- (IL-) 1*β*, IL-1ra, IL-6, IL-8, IL-10, and tumor necrosis factor- (TNF-) *α* using specific sandwich enzyme-linked immunosorbent assay (ELISA) according to the manufacturer's instructions (IL-1 *β*, IL-1ra, IL-10, and TNF-*α* were from R&D Systems, Minneapolis, MN; IL-6 and IL-8 from eBioscience, San Diego, CA, USA).

As a first stage of numerical analysis, data were sorted on the basis of subjects with (i) isolated ACL tear and (ii) ACL tear associated with meniscal tear, in order to establish the effects of the meniscal tear on knee synovial fluid cytokine patterns.

In the second stage of numerical analysis, data were sorted into three groups according to the time elapsed between trauma and samples collection, specifically (i) an acute group (between 0 and 48 h after injury), (ii) a subacute group (between 3 days and 3 months after injury), and (iii) a chronic group (more than 3 months after injury).

### 2.3. Statistical Analysis

Statistical analysis was performed using SPSS (version 22.0). Normality of data distribution was assessed by the Jarque-Bera test. The influence of growth plate maturity (i.e., open, closing, and closed), time from trauma (i.e., acute, subacute, and chronic), type of ACL injury (i.e., isolated, associated with meniscal tear), and Lysholm score (i.e., poor, fair, and good) on cytokine concentrations were assessed on the basis of generalized linear models with (i) cytokine concentrations as dependent variables; (ii) growth plate maturity, time from trauma, meniscal tear, and Lysholm score as predictive factors; and (iii) age and sex as covariates.

Correlations among various biochemical markers were assessed for significance using the nonparametric Spearman rank correlation coefficient test. For all statistical tests, a value of *p* < 0.05 was considered to be statistically significant.

**Figure 2:**
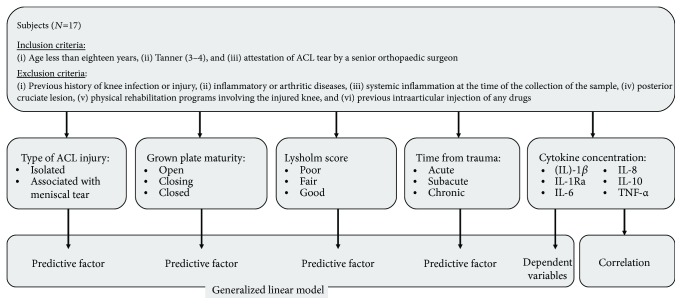
Flow chart with patient characteristics and inclusion and exclusion criteria of adolescent patients with ACL tear.

Figure 2 reports a flowchart with patients' characteristics, inclusion and exclusion criteria, cytokines evaluated, and statistical analysis.

## 3. Results

### 3.1. Subjects

Among the 17 patients, ACL tears were the result of (i) sports injuries (*n* = 14), (ii) vehicle-related collisions (*n* = 2), and (iii) miscellaneous recreational activity (*n* = 1). Patients were also sorted according to (i) acute ACL tear (*n* = 5), (ii) subacute ACL tear (*n* = 7), and (iii) chronic ACL tear (*n* = 5).

The mean Tegner score was 8 ± 1 (mean ± SD; range 6–10), and the Lysholm score at the moment of the arthrocentesis was 50.76 ± 26 (mean ± SD; range 5–88).

At the time of surgery, six patients (35%) were confirmed with an isolated ACL tear and eleven patients (65%) with an ACL tear associated with a meniscal injury.

Fifteen patients with intact articular surfaces and no sign of chondral pathology were graded as Outerbridge grade 0, and there was a single grade II patient. One patient with ACL tear associated with meniscal injury presented an osteochondral lesion with exposure of the subchondral bone that was classified as Outerbridge grade IV. Patient details are summarized in Table 1.

### 3.2. Effects of Time, Growth Plate Maturity, Meniscal Tear, and Clinical Score on Cytokine Expression in Adolescent ACL Tears

**Figure 3:**
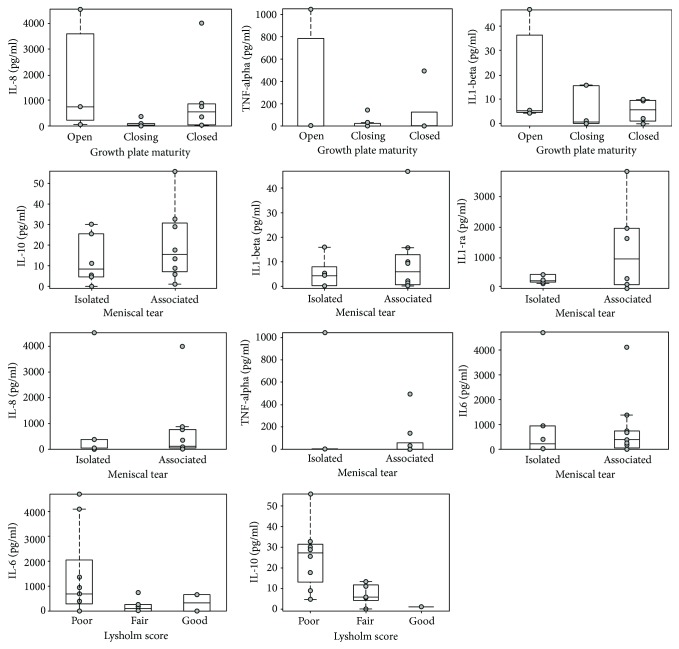
Modifications of cytokine levels in relation to growth plate maturity, meniscal tears, and Lysholm scores. Cytokine concentrations measured in the adolescent group (*n* = 17) are represented in the box plot. Grey dot represents cytokine concentrations of each single patient. In each box plot, the box is built within the third (upper bound) and first (lower bound) quartiles (i.e., 𝑄3, 𝑄1); the middle line represents the median. Whiskers represent data maximum (upper whisker) and minimum (lower whisker). Defined as data points below *Q*1–1.5 × (*Q*3 − *Q*1) or above *Q*3 + 1.5 × (*Q*3 − *Q*1).

The concentrations of six cytokines were measured by ELISA in the knee synovial fluid of all adolescent patients. The influence of growth plate maturity, time from trauma, meniscal tear, and the Lysholm score on cytokine levels are reported in Table 2 and Figure 3. Growth plate maturity shows a significant positive influence on IL-8 levels (*p* = 0.010), TNF-*α* levels (*p* = 0.026), and IL-1*β* levels (*p* = 0.013) (Table 2). High cytokine concentrations were found in synovial samples from patients with an open physis (Figure 3). The time elapsed from trauma did not significantly influence cytokine levels in the cohort of the analyzed adolescent patients (Table 2). A meniscal tear associated with ACL tear resulted in higher levels of IL-1ra (*p* = 0.031) and IL-1*β* (*p* = 0.012) (Table 2 and Figure 3). Moreover, the Lysholm score was negatively associated with IL-6 (*p* = 0.019) and IL-10 levels (*p* = 0.017), as poor results in the Lysholm scale were associated with higher levels of these cytokines (Figure 3).

### 3.3. Correlations between Cytokines in Adolescent Knees

In adolescent patients, cytokine levels in synovial fluid were not influenced by the time elapsed from trauma (Table 2); consequently, samples were considered as a single group. Bivariate correlations between synovial cytokines highlighted a positive correlation of IL-10 with IL-6 (*p* = 0.047, *r* = 0.538) and with IL-8 (*p* = 0.025, *r* = 0.596), whereas TNF-*α* was negatively correlated with IL-6 (*p* = 0.051, *r* = −0.513). Statistical analysis did not show any other correlation between cytokines.

## 4. Discussion

Different techniques have been used to treat ACL injuries in children and adolescent patients with conflicting results [31]. Describing the cytokine concentration profiles in a pediatric population with ACL tear may help clarify our understanding of factors influencing the outcome of a proposed surgical technique of ACL reconstruction and ACL reinsertion or suture.

In this study, we observed increased levels of IL-6 in patients with the worst clinical features (in terms of the Lysholm score). In previous scientific publications, high levels of IL-6 have been reported in adult patients with symptomatic meniscal tears, suggesting IL-6 could be involved in pain generation; IL-6 is also involved in the cartilage catabolic process and arthritis development [32]. High synovial levels of IL-6 and MCP-1 have been proposed as potential biomarkers or predictive factors for the clinical outcomes in knee arthroscopy in adults [32, 33], and the results that we have obtained in a cohort of a young population could support this hypothesis.

Interestingly, IL-10 levels were high in patients with the worst clinical features, and a positive correlation has been registered between IL-10 and IL-6. The potent inhibition on monocyte-macrophage function carried out by IL-10 allows considering this cytokine a protective factor; a positive correlation with IL-6 could suggest that anti-inflammatory mediators were released in an attempt to control inflammatory overshooting. Similarly, levels of IL-10 also correlate with those of IL-8 which exerts an intrinsic catabolic effect on chondrocytes [34, 35], confirming that IL-10 has a modulatory function in opposing IL-6 and IL-8 [36, 37]. A positive correlation between IL-8 and IL-10 was previously reported in adult patients with ACL tear [13].

Synovial levels of proinflammatory cytokines IL-8, TNF-*α*, and IL-1*β* were particularly elevated in patients with open femoral physis. A possible explanation is that the ACL tear induces an intra-articular catabolic response in adolescent patients greater than that reported for adult subjects. Further studies are needed to ascertain whether the stage of development of growth plates is a determinant of the extent of the intra-articular inflammatory response to traumatic knee injuries. Clinical and animal studies are required also to clarify the clinical implications.

To the best of our knowledge, this is the first study to analyze cytokine concentrations in injured knees of adolescent patients. Our results highlight high levels of both proinflammatory and anti-inflammatory cytokines. Previous studies measured cytokine concentrations only in adults. Considering our previous data about cytokine concentrations measured with the same methodological technique, we may attempt to identify some differences in the cytokine profile in adults compared to adolescents.

In particular, we notice that IL-6, IL-8 (proinflammatory), IL-10, and IL-1ra (protective factors) concentrations are higher in adolescents than in adults. IL-6 and IL-8 are thought to have an important role in cartilage degeneration and in the pathophysiology of osteoarthritis (OA) [38–40]. Levels of IL-1*β*, another proinflammatory cytokine, were indeed comparable with those in adult subjects [12]. On the other hand, the intra-articular concentration of IL-1ra, the natural antagonist of IL-1*β*, in our adolescent patients with ACL tears was higher than that previously measured in adults [13, 37]. This specific cytokine pattern characterized by high levels of modulatory cytokines (including proinflammatory) may suggest that in the adolescent population the inflammatory mechanisms could have a relevant role and affect healing processes [10, 13, 36].

In an adult population with ACL tear, time influenced cytokine levels in the synovial fluid [10, 14] since high levels of IL-1*β*, IL-6, IL-8, IL-1ra, and TNF-*α* were reported acutely after trauma [13]. Cytokine levels in the synovial fluid of adolescent patients may exhibit a time-dependent pattern, but our results suggest that in adolescent patients, intra-articular levels of TNF-*α*, IL-6, and IL-8 remained elevated for months after trauma. Further studies with greater numbers of pediatric patients are required to better characterize this phenomenon.

Some clinical studies started to show interesting results in preservative surgery for ACL [19, 41]. In selected pediatric proximal ACL tears, excellent results were obtained with a suture anchor ACL reinsertion [19]. However, in ACL midsubstance tear, this technique was not allowed. For pediatric ACL midsubstance tears, the possibility of a bridge-enhanced suture repair to preserve the remaining ACL tissue has been investigated [23, 41]. Ligament and meniscal repair in a pediatric population was explained by a different cellular activity and vascularization, but the repairing potential could be influenced also by the joint's inflammatory environment. TNF-*α* and IL-1 have been reported to play an inhibitory activity in meniscal repair processes in vitro; similarly, meniscal cell proliferation in vivo may be reduced by increased levels of proinflammatory cytokine [42–44]. Further studies are required to better understand the role of synovial inflammatory patterns in pediatric ACL repair.

We acknowledge the limitations of the present study: first, the small number of patients enrolled, due to the choice of stringent inclusion/exclusion criteria concerning age limits and the specific diagnosis of ACL tear, and second, the lack of assessment of cytokine correlation to postoperative clinical outcomes. Another potential limitation is the lack of positive and negative control groups; however, the absence of synovial samples from healthy skeletally immature knees was due to ethical reasons.

At last, we have considered only a selected group of cytokines, and we acknowledge that to better understand the complex biological environment in skeletally immature joints, more studies are required.

Our results suggest that the growth and the development of the skeleton could modulate the synovial cytokine milieu, leading to specific biochemical patterns in skeletally immature patients with ACL tear. We recommend more structured studies with larger sample sizes in order to enhance our understanding of the influence of age and bone maturity on cytokine patterns.

## Figures and Tables

**Table 1:** Preoperative evaluation of patient features. Diagnosis was confirmed intraoperatively. The Tegner scale varies from 0 to 10, where 0 represents sick leave or disability pension because of knee problems and 10 corresponds to participation in national and international elite competitive sports [28]. The Lysholm score varies from 0 to 100 and describes how knee pain affects everyday life. ACL: anterior cruciate ligament tear; MM: medial meniscus tear; LM: lateral meniscus tear. Growth plates were rated as described in Methods and Figure 1.

Number	Age	Sex	Mechanism of injury	Tegner	Lysholm	Side	Growth plate features	Diagnosis
1	17	M	Soccer	10	32	R	Closing	ACL
2	13	M	Soccer	7	24	L	Open	ACL
3	17	M	Rugby	8	5	L	Closing	ACL
4	17	M	Soccer	10	65	R	Closing	ACL
5	16	M	Recreational injury	6	68	R	Closing	ACL
6	13	F	Basketball	9	80	L	Open	ACL
7	16	M	Soccer	9	32	R	Closed	ACL + LM
8	14	M	Vehicle-related collision	9	35	R	Open	ACL + LM
9	16	M	Basketball	9	11	R	Closing	ACL + LM
10	17	M	Soccer	9	75	R	Closed	ACL + LM
11	16	M	Basketball	9	85	R	Closing	ACL + MM
12	17	M	Soccer	7	65	R	Closed	ACL + MM
13	14	M	Basketball	6	88	R	Closing	ACL + MM + LM
14	17	M	Soccer	9	65	L	Closed	ACL + LM
15	17	M	Vehicle-related collision	6	62	R	Closing	ACL + LM
16	17	M	Soccer	9	27	L	Closed	ACL + MM + LM
17	14	F	Soccer	7	44	R	Closed	ACL + MM + LM

**Table 2:** Correlations between clinical characteristics and cytokine levels in the synovial fluid of adolescents. Growth plate maturity and diagnosis are reported in Table 1. Lysholm score was calculated as described in Methods. Time elapsed between trauma and sample collection was considered as follows: acute: 0–48 h after injury; subacute: 3 days–3 months; chronic group: >3 months.

	IL-6	IL-8	TNF-*α*	IL-10	IL-1*β*	IL-1ra
Growth plate maturity	0.506	**0.010** ^∗^	**0.026** ^∗^	0.558	**0.013** ^∗^	0.883
Time from trauma	0.139	0.086	0.265	0.384	0.791	0.166
Meniscal tear	0.371	0.689	0.751	**0.055**	**0.012** ^∗^	**0.031** ^∗^
Lysholm score	**0.019** ^∗^	0.338	0.979	**0.017** ^∗^	0.170	0.759

∗ indicates values of *p* that reached statistical significance as resulting from the generalized fitted model.
